# The effect of geriatric intervention in frail older patients receiving chemotherapy for colorectal cancer: a randomised trial (GERICO)

**DOI:** 10.1038/s41416-021-01367-0

**Published:** 2021-04-07

**Authors:** Cecilia Margareta Lund, Kirsten Kjeldgaard Vistisen, Anne Pries Olsen, Pernille Bardal, Martin Schultz, Troels Gammeltoft Dolin, Finn Rønholt, Julia Sidenius Johansen, Dorte Lisbeth Nielsen

**Affiliations:** 1 Department of Medicine, Copenhagen University Hospital, Herlev and Gentofte, Copenhagen, Denmark; 2 CopenAge, Copenhagen Center for Clinical Age Research, University of Copenhagen, Copenhagen, Denmark; 3 Department of Clinical Medicine, Faculty of Health and Medical Sciences, Copenhagen University, Copenhagen, Denmark; 4 Department of Oncology, Copenhagen University Hospital, Herlev and Gentofte Hospital, Copenhagen, Denmark; 5 Department of Physiotherapy and Occupational Therapy, Copenhagen University Hospital, Herlev and Gentofte, Copenhagen, Denmark; 6 Nutritional and Dietetic Research Unit, Copenhagen University Hospital, Herlev and Gentofte, Copenhagen, Denmark

**Keywords:** Geriatrics, Colorectal cancer

## Abstract

**Background:**

Older patients with colorectal cancer (CRC) experience chemotherapy dose reductions or discontinuation. Comprehensive geriatric assessment (CGA) predicts survival and chemotherapy completion in patients with cancer, but the benefit of geriatric interventions remains unexplored.

**Methods:**

The GERICO study is a randomised Phase 3 trial including patients ≥70 years receiving adjuvant or first-line palliative chemotherapy for CRC. Vulnerable patients (G8 questionnaire ≤14 points) were randomised 1:1 to CGA-based interventions or standard care, along with guideline-based chemotherapy. The primary outcome was chemotherapy completion without dose reductions or delays. Secondary outcomes were toxicity, survival and quality of life (QoL).

**Results:**

Of 142 patients, 58% received adjuvant and 42% received first-line palliative chemotherapy. Interventions included medication changes (62%), nutritional therapy (51%) and physiotherapy (39%). More interventional patients completed scheduled chemotherapy compared with controls (45% vs. 28%, *P* = 0.0366). Severe toxicity occurred in 39% of controls and 28% of interventional patients (*P* = 0.156). QoL improved in interventional patients compared with controls with the decreased burden of illness (*P* = 0.048) and improved mobility (*P* = 0.008).

**Conclusion:**

Geriatric interventions compared with standard care increased the number of older, vulnerable patients with CRC completing adjuvant chemotherapy, and may improve the burden of illness and mobility.

**Trial registration:**

ClinicalTrials.gov ID: NCT 02748811.

## Background

Comprehensive geriatric assessment (CGA) in the trajectory of cancer care is recommended to improve treatment outcomes for older patients with cancer.^1^ As the incidence of cancer increases with age^2^ and populations are getting older,^3^ the interest in CGA is increasing.^4^ Older patients are a heterogeneous group, ranging from fit to frail, with varying comorbidities and ability to tolerate chemotherapy.^5^ Thus, chronological age by itself should not be an exclusion criterion for adjuvant or palliative chemotherapy.^6–8^

Colorectal cancer (CRC) mortality has decreased during the last decade, although most markedly in younger patients.^9,10^ Adjuvant chemotherapy for 3–6 months after surgery for stage II/III colon cancer (CC)^11–15^ and rectal cancer^16^ improves disease-free survival (DFS) and overall survival (OS). Adjuvant treatment prolongs DFS in patients ≥70 years,^17^ and OS is higher in CRC patients >75 years receiving adjuvant chemotherapy than in patients receiving no treatment.^18–20^ For patients with metastatic CRC, median survival is only 10–11 months,^21^ mainly due to comorbidity in older patients and patients with poor performance status (PS) who do not receive treatment.

CGA is a multidisciplinary evaluation of an older individual’s comorbidity and medications, functional, social and nutritional status, physical performance and cognitive and emotional function.^22,23^ In a meta-analysis of randomised controlled trials (RCTs), CGA-based interventions during hospitalisation increased the likelihood of being alive and living at home 6 months after hospital discharge compared with standard care.^24^

In older patients with cancer, CGA can predict chemotherapy toxicity, morbidity and survival,^25–28^ and is recommended by the American Society of Clinical Oncology (ASCO).^1^ Geriatric assessment can predict survival in patients after CRC surgery^29^ and identify geriatric problems, leading to changes in chemotherapy-treatment strategy in up to 54% of patients.^30^ The G8 questionnaire is recommended as a sensitive screening tool^31^ that can predict survival and treatment-related complications.^32^ For patients with a G8 score ≤14, full CGA is recommended.^33^

The aim of this study was to investigate whether CGA-based interventions in vulnerable older patients with CRC could enhance the number of patients completing scheduled chemotherapy.

## Materials and methods

### Study design

Phase 3 RCT comparing CGA-based interventions with standard care in vulnerable, older patients undergoing chemotherapy for CRC.^34^

### Setting

The study was conducted at Departments of Oncology and Medicine, Copenhagen University Hospital, Herlev and Gentofte and Nordsjællands Hospital, Denmark.

### Participants

#### Inclusion criteria

Patients ≥70 years with stage II–IV CRC referred to the Department of Oncology for treatment with the adjuvant or first-line palliative/downstaging chemotherapy, a life expectancy ≥3 months and a ECOG (Eastern Cooperative Oncology Group) PS of 0–2,^35^ and assessed as vulnerable using the G8 questionnaire (G8 ≤ 14).^36^ All patients gave signed informed consent. Exclusion criteria: co-existing with other cancer within 5 years and participating in pharmaceutical trials.

The study was primarily designed to include patients receiving adjuvant chemotherapy after surgery for primary CRC. Fewer vulnerable patients than expected were offered or accepted chemotherapy, and fewer than expected accepted participating in a clinical trial (25% non-participants). Therefore, inclusion criteria were changed after 1 year to also include patients receiving downstaging, first-line palliative chemotherapy and adjuvant chemotherapy after surgical resection of metastatic CRC. Inclusion at Nordsjællands Hospital was low and closed after the inclusion of four participants.

### Randomisation

The participants were randomised 1:1 to CGA-based interventions or standard care. Patients were stratified after PS 0–1 vs. 2 and adjuvant vs. palliative/downstaging chemotherapy.

### Interventions

#### Oncological treatment

All patients received standard treatment (Supplementary Table S1) consisting of 3–6 months of adjuvant chemotherapy^15^ or first-line palliative/downstaging chemotherapy with various duration until disease progression (CT scan every 3 months), surgery, change in treatment or end of treatment due to severe adverse events (AE). The standard dose was based on body surface area (BSA), with a possible 25% primary dose reduction if concerns of toxicity at the first oncological assessment.

#### CGA for the intervention group

Patients in the intervention group received CGA at the start of chemotherapy, if possible before the start of treatment, but otherwise up to a few days after chemotherapy start. The CGA included medication review, assessment of comorbidity, psycho-cognitive function and nutritional, functional and physical status with appropriate interventions, including referral to a dietitian and physical exercise programme (for CGA and implemented interventions see Table 1 and Supplementary Material). CGA-based interventions were followed up after 2 months or more frequently if needed.

**Table 1:** The comprehensive geriatric assessment, results and interventions.

Domain	Assessment and screening tool				Possible interventions	Interventions implemented	
		Cut-off	Score	*n* (%)			*n* (%)
Comorbidity	CIRS-G	–	0–4 5–7	11 (15) 31 (44)	Optimising treatment Referrals to exams/other departments	Referrals	23 (32)
	Review of medical records		≥8	29 (41)			
	Clinical examination						
	Patient interview						
Medication review	No. of medications/polypharmacy	–	0–4	43 (61)	Discontinuation	Changes in medication	44 (62)
			≥5	28 (39)	Prescription		
	START/STOP criteria				Change in dosage		
Cognitive function	MMSE	≤23/30	24–30	71 (100)	Further evaluation	Cognitive evaluation	1 (1.4)
			0–23	0 (0)	Referral/medication		
Psychological function	GDS	≥ 6/ 15	0–5	67 (9)	Assessment of possible depression	Medical treatment	2 (2.8)
			≥6	4 (6)		Referrals	2 (2.8)
Nutritional status	MNA-based local nutritional screening	Weight loss ≥5%	0–5	18 (25)	Nutritional supplements	Referral: GERICO dietitian	36 (51)
			≥5	53 (75)	Referral to dietitian^a^		
Physical function	Gait speed 10 m	>1 m/s	0–1	37 (52)	Referral to the exercise programme^b^	Referral: GERICO exercise programme	28 (39)
			>1	32 (45)			
	Handgrip strength (Jamar Dynamometer)	<♀ 20 kg	below	35 (49)	Referral to the exercise training programme^b^	Referral: GERICO exercise programme	28 (39)
		<♂ 30 kg	above	36 (51)			
Functional status	Katz ADL	<6	6	62 (87)	Initiation of home care	Initiation of social support	2 (2.8)
	(In)dependence		0–5.5	9 (13)	Occupational therapy assessment	Occupational therapy	2 (2.8)
	FAQ IADL	>1	0	48 (68)	Initiation of home care	Initiation of home care	2 (2.8)
	(In)dependence		≥1	23 (32)	Transport arrangement		
Laboratory parameters	TSH, cobalamin, folate, albumin, vitamin D	Normative values	Normal	51 (72)	Treat deficiencies/control blood samples	Deficiencies treated	20 (28)
			abnormal	20 (28)			

*ADL* activities of daily living, *CIRS-G* Critical Illness Rating Score—Geriatrics, *FAQ IADL* frequently asked questions instrumental activities of daily living, *GDS* Geriatric Depression Scale, *MMSE* minimal mental state examination, *MNA* minimal nutritional assessment.

^a^A personalised nutritional plan based on the patient’s taste preferences and a telephone follow-up call after 1 month, and 3 months after chemotherapy discontinuation.

^b^The exercise programme included 24 supervised exercise sessions preferably twice weekly at the hospital and home exercise once weekly. The supervised exercise was a 1-h training programme including 15 min of warming up with aerobic and balance training, 30 min of resistance training, and finally 15 min of supervised relaxation.

Co-existing health problems among controls were assessed by either an oncologist or general practitioner.

### Data collection, measures and outcomes

Collected data included demographics, comorbidities, medication, date of diagnosis, recurrence, progression and survival. Received chemotherapy, delays, AE (European Organization for Research and Treatment Cancer (EORTC) Common Terminology Criteria (CTC) version 4.0), weight and PS (including self-reported PS) were registered for all patients at every treatment cycle. For patients in the control group, information about weight loss or functional decline prior to chemotherapy was collected retrospectively from medical charts.

The patients participated in the study until planned treatment ended or occurrence of disease recurrence (adjuvant group), disease progression (palliative group), resection of metastases (downstaging group) or treatment stopped due to AEs or poor PS.

EORTC quality-of-life (QoL) questionnaires (QLQ) QLQ C30^37^ and QLQ ELD-14^38^ were completed in all patients prior to randomisation, at the oncological appointment prior to chemotherapy after 2 months, and at the end of treatment (at the oncological appointment prior to the last cycle of adjuvant chemotherapy (3 or 6 months) or in case of progression (palliative setting)) or at early discontinuation. QoL domains range from zero to 100, where a high score indicates a higher function for functional scores and a higher grade of symptom burden for symptom domains. For patients in the exercise programme, a physical test battery, including 30-s chair-stand test (CST), 30-s arm-curl test, leg-press maximum weight and climbing-stair test, was performed before and after 24 exercise sessions.

Blinding of oncologists was not possible, as the results of the geriatric assessment were included in the patients’ medical charts.

The primary outcome was the completion of planned chemotherapy without later dose modifications or delays (oxaliplatin not included). The primary outcome was assessed by a blinded oncologist. Secondary outcomes were dose reductions, treatment delays, adverse events and prognosis (DFS, PFS, and OS and CRC mortality).

### Statistical power and analyses

Planned treatment is usually completed in ~50% of patients.^19^ No prior study has assessed the effect of the geriatric intervention on chemotherapy completion, but completion was assumed to increase to 75% after the geriatric intervention. With 140 patients included, such an increase would be detected with a probability (power) of 87% at a 5% significance level.

Categorical variables were analysed using a chi-square test. The result of the exercise programme was analysed with a paired sampled *t* test. Dose intensity was defined as a cumulative given dose compared to a standardised total dose per week, and differences between the two groups were analysed with the Wilcoxon test.

Baseline QoL data were presented as means and standard deviations, and differences in mean change over time between the groups were analysed using the Wilcoxon test.

DFS was defined as the time from randomisation to disease recurrence or death, PFS as time from randomisation to disease progression or death and OS as time from randomisation to death of any cause. Survival was estimated with the Kaplan–Meier estimator and compared with a log-rank test as hazard ratios (HRs) and 95% confidence intervals (CIs).

All analyses were performed on complete cases only. All statistical analyses were performed using the statistical software package R, version 3.5.2^39^, with a significance level of 5%.

## Results

**Fig. 1:**
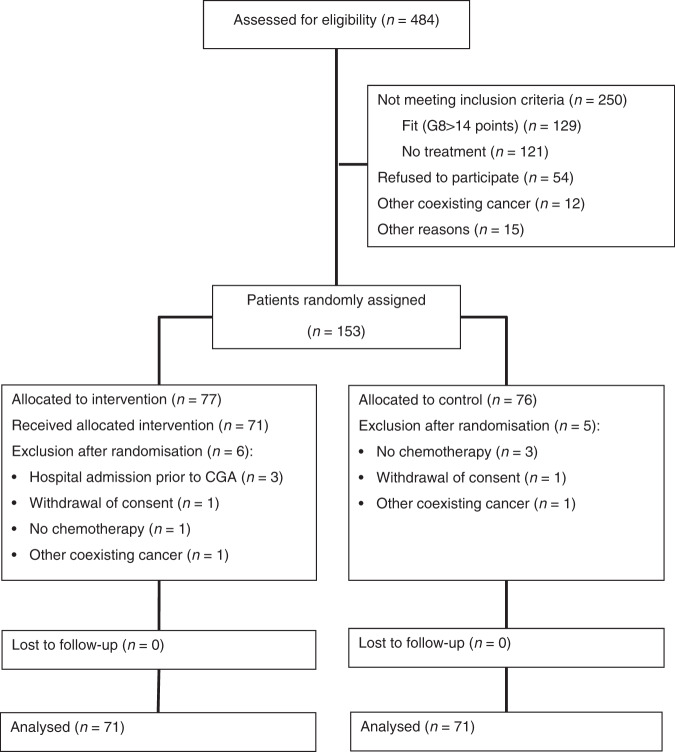
Screening and inclusion in the GERICO study. Totally 484 patients were assessed and 153 patients were found eligeble for the study.

From April 2015 to September 2019, 484 patients were assessed for eligibility (Fig. 1). In total, 54 patients did not meet inclusion criteria, since they were found to be fit and not vulnerable according to the G8 screening (*n* = 129) or the patients did not receive chemotherapy (*n* = 121). Fifty-four patients did not want to participate, and 12 patients were excluded due to other coexisting cancer or other reasons (*n* = 15). Of the 153 patients included, 11 patients were later excluded (withdrawal of consents (*n* = 2), treatment for coexisting cancer (*n* = 2), patients did not start chemotherapy (*n* = 4) and patients were hospitalised due to toxicity and discontinued chemotherapy prior to CGA (*n* = 3)). For baseline characteristics of the 142 included patients, see Table 2. Due to the heterogeneity caused by different treatment settings, analyses were performed in all patients and in two subgroups: patients who received adjuvant treatment after surgery for primary non-metastatic CRC (*n* = 77, intervention group (I) (*n* = 40), control group (C) (*n* = 37)), and patients who received palliative treatment for metastatic disease (no downstaging treatment) (*n* = 44, I (*n* = 22), C (*n* = 22)). Patients receiving chemotherapy after resection of metastases (*n* = 15) and patients who had received downstaging chemotherapy (*n* = 6) were not included in sub-group analyses.

**Table 2:** Baseline characteristics.

Characteristics		Intervention group, *N* = 71, *n* (%)	Control group, *N* = 71, *n* (%)
Age	Median (range)	75 (70–85)	75 (70–88)
Sex	Men	43 (61)	38 (54)
	Women	28 (39)	33 (46)
PS	0	32 (45)	34 (48)
	1	32 (45)	32 (45)
	≥2	7 (10)	5 (7.0)
Civil status	Single	26 (37)	22 (31)
	Living together	45 (63)	49 (69)
BMI	Median (range)	25 (16.8–37.2)	23.3 (16.5–33.6)
Comorbidity	0–4	11 (15)	20 (28)
CIRS-G score	5–7	31 (44)	26 (37)
	≥8	29 (41)	25 (35)
G8 score	Median (range)	12 (7–14)	12 (6–14)
Number of medications	0	2 (3)	6 (8.4)
	1–3	33 (47)	24 (34)
	4–5	19 (28)	22 (31)
	6+	17 (24)	19 (27)
Treatment setting	Adjuvant	40 (56)	37 (52)
	Palliative	22 (31)	22 (31)
	Downstaging	7 (10)	8 (11)
	Adjuvant after metastatic surgery	2 (3)	4 (5.6)
Start dose	Full	27 (38)	30 (42)
	Primary dose reduction	44 (62)	41 (58)
Tumour location	Right colon	32 (45)	31 (44)
	Left colon	25 (35)	29 (41)
	Rectum	14 (20)	11 (16)
CT regimen length adjuvant setting	3 months	0 (0)	3 (8)
	6 months	4 (100)	34 (92)
CT regimen (all patients)	Capecitabine/5-FU	21 (29.6)	26 (37)
	Capeox/Folfox	35 (49.3)	23 (32)
	Capiri/Folfiri	15 (21.1)	22 (31)
CT regimen	Capecitabine/5-FU	15 (37.5)	19 (51)
Adjuvant setting	Capeox/Folfox	25 (62.5)	18 (49)
CT regimen	Capecitabine/5-FU	6 (27.3)	5 (23)
Palliative setting	Capeox/Folfox	4 (18.2)	2 (9.1)
	Capiri/Folfiri	12 (54.5)	15 (68)
MSI	MSI stable	56 (79)	57 (80)
	MSI unstable	9 (13)	11 (16)
	Not known	6 (8)	3 (4.2)
KRAS status	Mutation	39 (55)	26 (37)
	No mutation	28 (40)	44 (62)
	Not known	4 (6)	1 (1)
NRAS status	Mutation	3 (4)	4 (5.6)
	No mutation	58 (82)	52 (73)
	Not known	10 (14)	15 (21)
BRAF status	Mutation	16 (23)	12 (17)
	No mutation	45 (63)	44 (62)
	Not known	10 (14)	15 (21)

*BMI* body mass index, *CT* chemotherapy, *MSI* microsatellite instability, *PS ECOG* performance status.

### Geriatric interventions and quality of life

Interventions were needed in 92% of patients in the intervention group (88% of patients in the adjuvant group and in 100% of palliative patients). The most frequently implemented interventions were changes in medication (62%), nutritional therapy (51%) and exercise (39%) (Table 1). The need for nutritional therapy and exercise was most pronounced in the palliative group (64% vs. 43% and 50% vs. 38%). None of the patients had cognitive impairment (mini-mental state examination (MMSE) below the cut-off <24) and 6% had possible depression.

#### Physiotherapy

Fifty-one of 71 patients (72%) in the intervention group performed below cut-off in at least one of the physical screenings and were considered in need of physiotherapy. Of the 28 patients who accepted referral to the exercise programme, 24 agreed to postinterventional tests. The exercise programme was found to significantly increase physical strength in all four tests (Supplementary Table S2), at a level known to be a clinically important difference.^40^

Self-reported physical decline assessed by ECOG PS was seen in both groups (25% vs. 35%, *P* = 0.201). In the adjuvant setting, there was no difference (28% vs. 32%, *P* = 0.637), but in the palliative group, fewer interventional patients experienced a physical decline (18% vs. 50%, *P* = 0.026). Sixteen (23%) control patients were referred to municipal physiotherapy. Patients receiving therapy (intervention and controls combined) had less decline in PS than patients deemed in need of physiotherapy, but not exercising (18.5% vs. 31.8%, *P* = 0.128) with a significant difference among adjuvant patients (10.3% vs. 34.6%, *P* = 0.048). Also when analysing changes in QoL from start to 2 months, mobility was significantly worsened in controls compared with interventional patients during the study period (−8.39 standard deviation (SD) 24.69 vs. −0.43 SD 17.46, *P* = 0.008) (Supplementary Table S3), and burden of illness decreased more in the intervention than in the control group (−5.13 SD 25.68 vs. 4.67 SD 20.77, *P* = 0.048).

#### Nutritional therapy

Fifty-three (75%) patients in the intervention group had weight loss >5% prior to chemotherapy and were considered at risk of malnutrition, and 36 patients (51%) accepted dietitian referral. Of patients treated with palliative chemotherapy, 23% refused nutritional consultation. Among controls, 45 patients (63%) were at risk of malnutrition and 8 patients (11%) were referred to a dietitian.

Further significant weight loss, defined as >2.5% during treatment^41^, was seen in 15% of patients in the intervention group and in 24% of controls (*P* = 0.206).

### Primary endpoint (completion of planned chemotherapy)

**Fig. 2:**
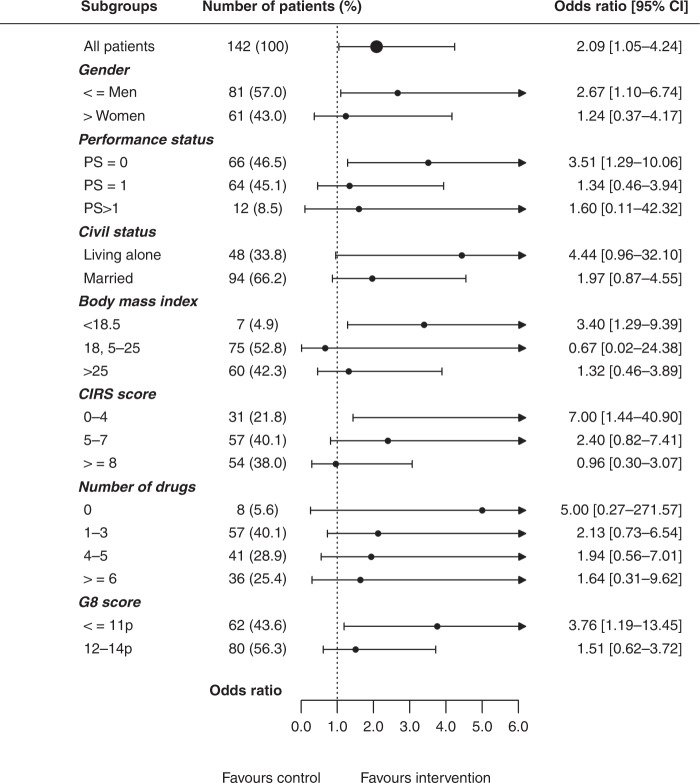
Association between baseline characteristics and completion of planned chemotherapy. CIRS Critical Illness Rating Scale, PS performance status.

More patients in the intervention group completed planned chemotherapy without further dose reductions or delays than in the control group (45% vs. 28%, *P* = 0.0366) (Table 3). The difference was most prominent in patients in the adjuvant setting (*P* = 0.0097), whereas no significant difference was seen in the palliative group (*P* = 0.751). Associations between baseline characteristics and the effect of the geriatric intervention on completion of scheduled chemotherapy are shown in Fig. 2. The beneficial effect of CGA was mainly found in patients with G8 score ≤11 (odds ratio OR 3.76, 95% confidence interval (CI) 1.19–13.45).

**Table 3:** Outcomes: chemotherapy received, dose reduction and delays.

Variable	All patients, *N* = 142			Adjuvant setting, *N* = 77			Palliative setting, *N* = 44		
	I *N* = 71, *n* (%)	C *N* = 71, *n* (%)	*P*	I *N* = 40, *n* (%)	C *N* = 37, *n* (%)	*P*	I *N* = 22, *n* (%)	C *N* = 22, *n* (%)	*P*
Completed planned treatment (primary endpoint) initial dose in all planned cycles	32 (45)	20 (28)	**0.0366**	20 (50)	8 (22)	**0.0097**	7 (32)	8 (36)	0.751
Reduced start dose	44 (62)	41 (58)	0.732	23 (58)	16 (43)	0.211	15 (68)	19 (86)	0.150
Reduction of chemotherapy during treatment	20 (28)	32 (45)	**0.037**	10 (25)	21 (57)	**0.005**	8 (36)	7 (32)	0.750
Treatment delay	25 (35)	24 (34)	0.860	8 (20)	15 (41)	**0.049**	13 (59)	6 (27)	**0.033**
Received initial dose in all given cycles	46 (65)	30 (42)	**0.007**	25 (63)	11 (30)	**0.004**	14 (64)	12 (59)	0.540
Received all planned cycles	41 (58)	39 (55)	0.735	26 (65)	21 (57)	0.459	10 (45)	11 (50)	0.763
	**Median (range)**		***P***	**Median (range)**		***P***	**Median (range)**		***P***
Dose intensity: capecitabine/5-FU (%)	84 (35–117)	89 (11–117)	0.724	89 (67–117)	95 (47–117)	0.662	93 (35–112)	75 (11–100)	0.343
Dose intensity: oxaliplatin (%)	75 (22–169)	87 (31–130)	0.134	80 (22–169)	87 (31–92)	0.304	78 (49–103)	76 (76–76)	1.000
Dose intensity: irinotecan (%)	65 (27–116)	66 (4–110)	0.883	–	–	–	67 (27–116)	63 (4–104)	0.240
% of planned cycles received capecitabine/5-FU	100 (8–100)	100 (8–100)	NA	100 (8–100)	100 (8–100)	NA	89 (13–100)	92 (13–100)	0.763
% of planned cycles received oxaliplatin	58 (8–100)	63 (8–100)	0.825	50 (8–100)	50 (8–100)	0.888	89 (25–100)	100 (100–100)	1.000
% of planned cycles received irinotecan	93 (60–100)	96 (25–100)	0.751	–	–	–	91 (60–100)	90 (25–100)	1.000
Duration of chemotherapy (weeks)	22.1 (0.3–43)	20.1 (0.1–53)	0.905	22.6 (0.3–27)	22.4 (0.3–29)	0.855	17.2 (0.3–43)	21.6 (0.1–52)	0.675

*I* intervention, *C* control, *5-FU* 5-flourouracil.

Bold values indicate significant differences between the intervention and control group.

### Dose reductions, delays and received chemotherapy

Start doses were reduced in 60% of all patients with no difference between the intervention and control group (Table 3). Secondary dose reductions occurred more rarely in the intervention group (28% vs. 45%, *P* = 0.037). More patients in the intervention group received the planned dose in all given cycles of chemotherapy (65% vs. 42%, *P* = 0.007). There were no differences between the two groups regarding the received median dose intensity or a number of cycles for capecitabine, 5-fluorouracil, oxaliplatin or irinotecan throughout the treatment. No difference was found in median treatment duration and delays occurred equally in both.

### Adverse events and hospitalisation

In total, 28% of patients in the intervention group experienced toxicity grade 3 or more compared with 39% among controls (*P* = 0.156). Most frequently, grade 3+ adverse events were infections (*n* = 11), cardiotoxicity (*n* = 10) and fatigue (*n* = 8). For all grade AEs, see Table 4. Hospitalisation during chemotherapy occurred with equal frequency in both groups (30% vs. 32%, *P* = 0.857) and 20% in the intervention group and 30% in the control group discontinued chemotherapy due to toxicity (*P* = 0.173).

**Table 4:** Adverse events in intervention and control patients, depending on chemotherapy regimen.

Chemotherapy regimen	1			2			3		
Toxicity	Intervention, *N* = 21	Control, *N* = 26	*P*	Intervention, *N* = 35	Control, *N* = 23	*P*	Intervention, *N* = 15	Control, *N* = 22	*P*
*All grades*									
Neuropathy	2 (10)	4 (15)	0.439	25 (71)	18 (78)	0.260	1 (7)	1 (45)	0.341
PPE	11 (52)	15 (58)	0.174	8 (23)	7 (30)	0.708	0	6 (27)	0.030
Infection	2 (10)	3 (12)	0.824	6 (17)	4 (17)	0.413	1 (7)	4 (18)	0.297
Fatigue	16 (76)	23 (88)	0.427	34 (97)	21 (91)	0.512	13 (87)	22 (100)	0.207
Diarrhoea	8 (38)	13 (50)	0.704	16 (46)	14 (61)	0.055	8 (53)	10 (45)	0.427
Nausea	7 (33)	10 (38)	0.930	21 (60)	12 (52)	0.912	10 (67)	16 (73)	0.187
Mucostomatitis	4 (19)	12 (46)	0.107	9 (26)	9 (39)	0.349	6 (40)	6 (27)	0.417
Neutropenia	0	0	NA	4 (11)	4 (17)	0.475	4 (27)	2 (9)	0.217
Thromboembolism	0	0	NA	2 (5.7)	0	0.506	0	0	NA
*Grades 1–2*									
Neuropathy	2 (9.5)	2 (7.7)	0.827	28 (80)	14 (61)	0.116	1 (6.7)	1 (4.5)	0.774
PPE	9 (43)	12 (57)	0.345	7 (20)	7 (30)	0.367	0	6 (27)	0.030
Infection	2 (9.5)	3 (12)	0.787	2 (5.7)	4 (17)	0.165	6 (40)	4 (18)	0.147
Fatigue	16 (76)	21 (81)	0.711	31 (89)	20 (87)	0.829	13 (87)	20 (91)	0.675
Diarrhoea	7 (33)	12 (46)	0.375	13 (37)	12 (52)	0.263	8 (53)	9 (41)	0.458
Nausea	7 (33)	10 (39)	0.674	19 (54)	11 (48)	0.658	10 (67)	12 (55)	0.478
Mucostomatitis	4 (19)	12 (46)	0.055	9 (26)	9 (39)	0.284	6 (40)	6 (27)	0.417
Neutropenia	0	0	NA	3 (8.6)	3 (13)	0.593	3 (20)	2 (9.1)	0.347
Thromboembolism	0	0	NA	1 (2.9)	0	0.414	0	0	NA
*Gade 3+*									
Neuropathy	0	2 (7.7)	0.199	1 (2.9)	4 (17)	0.062	0	0	NA
PPE	2 (9.5)	3 (12)	0.787	1 (2.9)	0	0.414	0	0	NA
Infection	0	0	NA	6 (17)	2 (8.7)	0.373	2 (20)	2 (9.1)	0.347
Fatigue	0	2 (7.7)	0.199	3 (8.6)	1 (4.3)	0.531	0	2 (9.1)	0.236
Diarrhoea	1 (4.8)	1 (3.8)	0.876	3 (8.6)	2 (8.7)	0.989	0	1 (4.5)	0.414
Nausea	0	0	NA	2 (5.7)	1 (4.3)	0.815	0	4 (18)	0.080
Mucostomatitis	0	0	NA	0	0	NA	0	0	NA
Neutropenia	0	0	NA	1 (2.9)	1 (4.3)	0.777	1 (6.7)	0	0.225
Thromboembolism	0	0	NA	1 (2.9)	0	0.414	0	0	NA

*NA* not applicable, *PPE* palmar-plantar erythrodysesthesia.

Note: Data presented as no. (%). Chemotherapy regimen; (1) capecitabine or 5-fluorouracil (+/÷ bevacizumab); (2) capecitabine or 5-fluorouracil + oxaliplatin; (3) capecitabine or 5-fluorouracil + irinotecan.

### Prognosis

The median follow-up time was 27 months (1–65 months). There was no difference between intervention and control patients in prognosis, DFS (adjuvant subgroup (*n* = 77), HR = 1.60, 95% CI 0.75–3.41), PFS (palliative subgroup (*n* = 44), HR = 0.91, 95% CI 0.48–1.71) or in OS (all patients (*n* = 142), HR = 1.13, 95% CI 0.68–1.87) and CRC-related mortality (all patients (*n* = 142), HR = 0.98, 95% CI 0.56–1.72) (Supplementary Fig. S1) (for intention-to-treat analyses, see Kaplan–Meier plots (Supplementary Fig. S2)).

## Discussion

Among vulnerable, older patients undergoing chemotherapy for stage II–IV CRC, we found that patients receiving CGA-based interventions were more likely to complete scheduled chemotherapy than patients receiving standard care. Furthermore, patients in the intervention group with CGA-based interventions were less likely to experience dose reductions and more patients received full-dose chemotherapy in all cycles, especially in the adjuvant group. This study is one of the first RCTs evaluating the effect of the geriatric intervention on chemotherapy completion rates in vulnerable, older people, a group of patients often underrepresented in clinical trials.^42^ Our findings are in line with a recent RCT by Kalsi et al. investigating the impact of the geriatric intervention on chemotherapy tolerance in 46 older patients with cancer.^43^ They found that patients receiving CGA were more likely to complete planned chemotherapy. The power calculation for this study was based on historical numbers of completion rates for patients above 70 years, which was ~50%. However, such numbers for frail or vulnerable older patients and a possible effect of CGA-based interventions were not to be found, as those studies had not yet been performed. Completion rates differed by only 17%, however, this constitutes a 60% relative completion improvement, achieved by a harmless intervention, by which the findings are still of clinical importance.

In this study, we found a relatively low rate of grade 3+ adverse events (28–39%), with no significant difference in line with Kalsi et al. who found grade 3+ toxicity rates of 43% in the intervention group and 52% among controls. In the larger-cluster RCT by Mohile et al. (*n* = 557)^44^ and the GAIN study (*n* = 600)^45^, geriatric interventions significantly reduced the generally higher rates of grade 3+ toxicity from 60 to 50%. We found that the QoL domain mobility and burden of illness improved after the geriatric intervention. The changes in QoL were small but were likely to be clinically important for patients according to Crosby et al.^46^ and Norman et al.^47^ who suggested a change of 0.2 and 0.5 standard deviations, respectively, as a useful threshold for discriminating minimal clinically important differences in changes in QoL. Snyder et al. reported on only minimal changes in EORTC-C30 scores for patients who simultaneously reported increased need of supportive care.^48^ However, QoL analyses are connected with uncertainties related to multiple testing and no domains were pre-defined as the main point of interest in the present study. Furthermore, minimal clinically important differences for ELD-14 have not been reported or defined, and this is why the findings should be interpreted with caution.^49^ On the other hand, the present results are in line with the INTEGRATE pilot study; geriatric intervention was also found to increase physical function and several other QoL domains compared with patients receiving usual care.^50^

In this study, reduced start doses were given to a considerably large proportion of all patients, due to concerns of toxicity. However, there was no difference between the intervention and control group, which is why this is unlikely to have had an impact on the results. It is unclear whether older patients should receive the highest possible dose of chemotherapy or reduced doses. High-dose intensities of chemotherapy have been associated with longer survival and decreased cancer mortality in patients with metastatic CRC.^51^ However, chemotherapy and toxicity risk must be carefully balanced, to avoid over- and undertreatment and thereby retain the functional capacity and QoL, possibly better done if integrating geriatric domains before treatment decisions.^52^

In the present RCT, all patients did not accept some of the offered interventions. Thus, a study design where all patients had received exercise and nutritional therapy might have led to more significant results. There seemed to be a difference in patients’ willingness to undertake interventions, most frequently due to loss of energy and not wanting more hospital appointments; the patients treated with adjuvant chemotherapy were more likely to accept the suggested interventions. This may explain why the positive results were more prominent among patients in the adjuvant setting. The risk of malnutrition was seen in the majority of patients, which is known to be associated with poor survival and decreased treatment completion in cancer patients.^53,54^ Not all patients at risk accepted dietitian referral and several controls were referred to the dietitian, which may have had an impact on the results.

The exercise was effective in the intervention group. Strength and physical capacity improved significantly in all four tests, which is of clinical importance.^40^ The CST mean improvement was three repetitions, which is known to be a clinically important difference in frail older patients.^55^ We found less subjective feelings of physical decline in the intervention group, most marked in the palliative group. Early rehabilitation is recommended to regain or maintain functional status and increase tolerance to cancer treatment.^56^

None of the patients performed below cut-off in cognitive screening. Most patients were well-educated and MMSE might not be sensitive enough to detect mild cognitive impairments in patients with high cognitive function.^57^ Patients with clear signs of dementia were not offered chemotherapy and therefore not screened for this study. Psychological screening detected possible depression in only 6%, which is lower than that seen in previous data, suggesting depressive symptoms in ~30% of older patients with cancer.^58^ Our study population was generally independent. However, functional decline and the need for social services in the treatment trajectory should be assessed, especially among patients in the palliative setting.

The limitations include the single-centre design, and that the geriatric assessments were performed by a geriatrician and not a multidisciplinary team. Due to ethical considerations, no geriatric baseline measures, including weight loss and physical decline, were applied to the control group; thus, no comparisons could be made regarding CGA. Due to lower recruitment rates than expected, the inclusion criteria were broadened after 1 year, making the study population more heterogeneous. Further limitations include lack of predefined QoL domains of interest, in combination with the non-patient-centred primary endpoint. Treatment aims and goals are likely to be different in adjuvant and palliative treatment settings, where maintenance of physical capacity or change in a QoL domain might have been more patient-relevant outcomes, especially among palliative patients.

During the recruitment period, international guidelines for adjuvant treatment of CRC patients changed and the duration was shortened from 6 to 3 months for some patients,^15^ which was not included in the randomisation strategy. Only patients in the control group were coincidentally scheduled for the more manageable 3 months’ treatment. Nevertheless, more patients in the intervention group completed adjuvant chemotherapy.

Our study included vulnerable patients, but not all patients needed any type of intervention, and for patients with G8 score ≤11, geriatric interventions were especially beneficial. Patients who were assessed too frail for chemotherapy and therefore not included in our study might have had an even greater need of CGA. Future research should focus on patient-centred outcomes as QoL or functional status, also in patients deemed too frail for chemotherapy and whether CGA-based interventions can improve health status and reassessment for active treatment.

Finally, the follow-up time was short, and our trial was not powered (sample size too small) to evaluate secondary survival outcomes, and we found no difference in DFS, PFS and OS between the intervention and control groups.

In conclusion, this RCT demonstrates the benefit of geriatric interventions for older, vulnerable patients with CRC. Geriatric interventions compared with standard care increased the number of older, vulnerable patients with CRC completing scheduled adjuvant chemotherapy with no increase in adverse events. Geriatric interventions may also improve the burden of illness and mobility; however, larger studies are needed to confirm the beneficial findings. Furthermore, patients in the palliative setting reported less functional decline after geriatric interventions than patients receiving standard care. Exercise significantly improved muscle strength and physical capacity. Geriatric interventions, including supervised exercise, can be recommended to improve treatment outcomes in older, vulnerable patients receiving chemotherapy for CRC.

## Supplementary information

Supplementary files

## Data Availability

For data supporting the results of this study, contact the corresponding author.
