# Supplementary material for: The effect of geriatric intervention in frail older patients receiving chemotherapy for colorectal cancer: a randomised trial (GERICO)
Source: Br J Cancer. 2021 Apr 7;124(12):1949–58. doi: 10.1038/s41416-021-01367-0 (PMC8185087; doi:10.1038/s41416-021-01367-0)
Supplement: Supplementary file 1 — Supplementary files [file 41416_2021_1367_MOESM1_ESM.docx]

**Table S1.** Chemotherapy in the GERICO study

Standardized oncological treatment and follow-up

| **Adjuvant setting** | | | |
| --- | --- | --- | --- |
| **Treatment** | **Drug** | **Dose** | **Frequency** |
| Capecitabine | Capecitabine | 2000 mg/m² oral daily for 14 days | Every 3 weeks* |
| 5-FU | 5-FU  Calcium folinate | Bolus: 400 mg/m² IV  Infusion 46 h: 2400 mg/m² IV  400 mg/m² iv. | Every 2 weeks** |
| Capeox | Capecitabine  Oxaliplatin | 2000 mg/m² oral daily for 14 days  130 mg/m² IV | Every 3 weeks* |
| Folfox | 5-FU  Calcium folinate  Oxaliplatin | Bolus: 400 mg/m² IV  Infusion 46 h: 2400 mg/m² IV  400 mg/m² IV  85 mg/m² IV | Every 2 weeks** |

*Maximum 4 or 8 series; **Maximum 6 or 12 series.

Abbreviations: 5-FU 5-Flourouracil; IV intravenous

Follow-up program in adjuvant setting: CT scans 1 and 3 years and colonoscopy 5 years after surgery

| **Palliative setting** | | | |
| --- | --- | --- | --- |
| **Treatment** | **Drug** | **Dose** | **Frequency** |
| Capecitabine | Capecitabine | 2000 mg/m² oral daily for 14 days | Every 3 weeks * |
| 5-FU | 5-FU  Calcium folinate | Bolus: 400 mg/m² IV  Infusion 46 h: 2400 mg/m² IV  400 mg/m² IV | Every 2 weeks ** |
| Capeox | Capecitabine  Oxaliplatin | 2000 mg/m² oral daily for 14 days  130 mg/m² IV | Every 3 weeks * |
| Folfox | 5-FU  Calcium folinate  Oxaliplatin | Bolus: 400 mg/m² IV  Infusion 46 h: 2400 mg/m² IV  400 mg/m² IV  85 mg/m² IV | Every 2 weeks *** |
| Irinotecan | Irinotecan | 180 mg/m² IV | Every 2 weeks **** |
| Capiri | Capecitabine  Irinotecan | 1600 mg/m² oral daily for 14 days  200 mg/m² IV | Every 3 weeks * |
| Folfiri | 5-FU  Calcium folinate  Irinotecan | Bolus: 400 mg/m² IV  Infusion 46 h: 2400 mg/m² IV  400 mg/m² IV  180 mg/m² IV | Every 2 weeks **** |
| Irox | Irinotecan  Oxaliplatin | 165 mg/m² IV  85 mg/m² IV | Every 2 weeks **** |

*optional addition of bevacizumab bolus 7.5 mg/m² IV; **optional addition of bevacizumab bolus 5 mg/m² IV

***optional addition of bevacizumab bolus 5 mg/m² IV; irinotecan bolus 165 mg/m² IV; cetuximab bolus 500 mg/m² IV or panitumumab 6 mg/kg IV **** optional addition of bevacizumab bolus 5 mg/m² IV, cetuximab bolus 500 mg/m² IV or panitumumab 6 mg/kg IV. Abbreviations: 5-FU 5-Flourouracil; IV intravenous

Follow-up program in the palliative setting: CT scans every 3 months.

**Definition of planned treatment for patients in palliative setting:** Chosen regimen, at the individual start dose until progression or stable disease/ complete remission of visible tumor (followed by CT scans). Thus, delays / skipping of cycles or dose reductions (not oxaliplatin) were deviations from planned treatment. For patients receiving downstaging chemotherapy, treatment continued until surgery or progression, with reevaluation with CT scan every second month.

**Dose reductions for all patients:** Reductions in one or several agents (not oxaliplatin) compared with the initiated start dose. Thus, it could be total withdrawal of one agent in a combination regimen or reduced doses of one or several agents/ reduced dose in a single agent therapy. Dose reductions were defined as a conscious action by the treating oncologist due to side effects.

**Table S2**. Results of the exercise program

| **Test** | ***N*** | **Start**  **Mean (SD)** | **12 weeks**  **Mean (SD)** | **Change**  **Mean (SD)** | ***P*** | **Size effect** |
| --- | --- | --- | --- | --- | --- | --- |
| Chair to stand (30s) | 24 | 10.83 (3.8) | 13.75 (4.2) | 2.92 (3.7) | <.001 | 0.77 |
| Leg press max. kg | 20 | 73.00 (25.2) | 91.00 (34.8) | 18.00 (15.4) | <.001 | 0.86 |
| Arm curl (30s) | 24 | 12.75 (3.1) | 16.21 (3.7) | 3.46 (3.45) | <.001 | 1.11 |
| Climbing stairs (steps/s) | 23 | 1.91 (0.5) | 2.28 (0.7) | 0.36 (0.39) | <.001 | 0.67 |

SD standard deviation

**Table S3.** Quality of life for patients in the intervention and control group

Patients in all treatment settings (EORTC QLQ C30 and ELD 14)

|  | **Baseline** | | | | | **Change 0-2 months** | | | | | **Change 2-6 months** | | | | | **Change 0-6 months** | | | | |
| --- | --- | --- | --- | --- | --- | --- | --- | --- | --- | --- | --- | --- | --- | --- | --- | --- | --- | --- | --- | --- |
| Domains |  |  |  |  | Between groups |  |  |  | Within group | Between groups |  |  |  | Within group | Between groups |  | | | Within group | Between groups |
|  | *N* | Mean | SD | Median | *P** | *N* | Mean change | SD | *P* | *P** | *N* | Mean change | SD | *P* | *P** | *N* | Mean change | SD | *P* | *P** |
| QLQ-C30 |  |  |  |  |  |  |  |  |  |  |  |  |  |  |  |  |  |  |  |  |
| Physical function | I: 71  C: 71 | 75.3  80.6 | 19.6  19.5 | 80.0  80.0 | .066 | I: 67  C: 68 | 0.30  -3.92 | 15.41  19.49 | .875  .102 | .433 | I: 52  C: 51 | 1.15  -1.96 | 14.86  10.61 | .578  .193 | .225 | I: 53  C: 51 | 1.76  -2.88 | 18.17  18.87 | .484  .282 | .203 |
| Emotional function | I: 71  C: 71 | 82.9  80.4 | 14.08  18.9 | 83.3  83.3 | .776 | I: 67  C: 66 | 2.24  5.30 | 12.44  15.72 | .146  .008 | .304 | I:52  C: 50 | -0.16  -4.83 | 11.84  12.50 | .923  .009 | .132 | I: 53  C: 51 | 1.41  3.59 | 13.65  16.77 | .454  .132 | .700 |
| Role function | I: 71  C: 71 | 67.6  69.8 | 28.6  31.1 | 66.7  83.3 | .482 | I: 65  C: 63 | -18.21  -9.79 | 30.15  29.73 | <.001  .011 | .175 | I: 52  C: 48 | 18.91  10.07 | 35.86  31.65 | <.001  .032 | .086 | I: 52  C: 51 | -2.56  0.65 | 31.38  40.41 | .558  .909 | .644 |
| Social function | I: 71  C: 71 | 86.4  89.9 | 21.9  18.8 | 100.0  100.0 | .320 | I: 67  C: 66 | -19.4  -20.45 | 29.24  23.71 | <.001  <.001 | .800 | I: 53  C: 50 | 19.81  16.00 | 24.47  23.56 | <.001  <.001 | .407 | I: 53  C: 51 | -1.89  -2.94 | 23.94  18.78 | .569  .268 | .961 |
| Cognitive function | I: 71  C: 71 | 89.9  88.0 | 12.7  18.1 | 100.0  100.0 | .927 | I: 68  C: 68 | -21.57  -18.14 | 20.78  22.99 | <.001  <.001 | .211 | I: 53  C: 51 | 16.35  17.97 | 20.01  18.51 | <.001  <.001 | .881 | I: 53  C: 51 | -5.03  0.65 | 18.08  14.51 | .048  .749 | .159 |
| Fatigue | I: 71  C: 71 | 35.4  34.1 | 20.4  24.9 | 33.3  33.3 | .613 | I: 67  C: 66 | 2.82  4.21 | 20.68  22.54 | .269  .134 | .422 | I: 52  C:48 | -0.43  2.78 | 25.37  16.45 | .904  .248 | .678 | I: 53  C: 50 | 3.14  6.67 | 21.94  23.98 | .302  .055 | .346 |
| Nausea | I: 71  C: 71 | 3.3  5.6 | 11.1  13.5 | 0.0  0.0 | .312 | I: 68  C: 68 | 3.68  7.11 | 15.99  20.83 | .062  .006 | .341 | I: 53  C: 51 | 2.83  0.33 | 16.90  13.94 | .228  .868 | .681 | I: 53  C: 51 | 6.92  4.90 | 20.26  18.04 | .016  .058 | .498 |
| Pain | I: 71  C: 71 | 11.03  15.3 | 17.4  23.4 | 0.0  0.0 | .309 | I: 68  C: 67 | 4.90  -2.24 | 24.95  24.08 | .110  .449 | .071 | I: 53  C: 51 | -3.77  6.21 | 23.94  24.49 | .256  .076 | .077 | I: 53  C: 51 | 2.83  0.98 | 21.12  28.75 | .334  .809 | .490 |
| Global QoL | I: 71  C: 71 | 64.3  66.8 | 22.1  22.3 | 66.7  66.7 | .520 | I: 68  C: 68 | 0.37  -0.74 | 21.28  20.55 | .887  .769 | 1.000 | I: 53  C:51 | 3.30  -3.27 | 22.61  19.30 | .293  .232 | .232 | I: 53  C: 51 | 3.14  0.82 | 20.23  22.56 | .263  .797 | .669 |
| QLQ ELD 14 |  |  |  |  |  |  |  |  |  |  |  |  |  |  |  |  |  |  |  |  |
| Mobility | I: 71  C: 71 | 73.40  79.50 | 26.86  22.90 | 77.78  88.89 | .160 | I: 66  C: 68 | 1.01  -4.41 | 17.73  20.51 | .645  .081 | .225 | I: 51  C: 49 | -1.09  -6.80 | 18.76  20.64 | .680  .025 | .063 | I: 52  C: 49 | -0.43  -8.39 | 17.46  24.69 | .861  .021 | .008 |
| Burden of illness | I: 71  C: 71 | 46.94  42.48 | 26.92  28.00 | 33.33  33.33 | .343 | I: 67  C: 68 | 7.21  3.68 | 30.87  30.72 | .060  .327 | .746 | I: 52  C: 50 | -5.13  4.67 | 25.68  20.77 | .156  .119 | .048 | I: 52  C: 50 | 0.96  4.33 | 33.87  34.14 | .839  .374 | .750 |
| Future worries | I: 71  C: 71 | 38.18  38.18 | 30.03  27.90 | 33.33  33.33 | .874 | I: 66  C: 68 | -0.67  0.81 | 22.21  20.97 | .806  .749 | .747 | I: 50  C:49 | -2.67  -4.76 | 21.36  16.83 | .382  .053 | .741 | I: 51  C: 49 | -4.36  -3.17 | 27.49  23.35 | .263  .346 | .574 |
| Worries about others | I: 71  C: 71 | 29.81  31.46 | 29.54  26.51 | 33.33  33.33 | .522 | I: 65  C:68 | -2.82  -1.72 | 22.93  20.19 | .325  .486 | .818 | I: 50  C:50 | -6.67  -3.00 | 23.81  22.51 | .053  .351 | .600 | I: 52  C: 50 | -9.61  -6.33 | 31.72  21.79 | .033  .045 | .855 |

*** Wilcoxon rank sum test

C Control group; I Intervention group; SD Standard deviation; QoL Quality of life; QLQ Quality of Life Questionnaire

**Figure S1.**

a. Disease-free survival


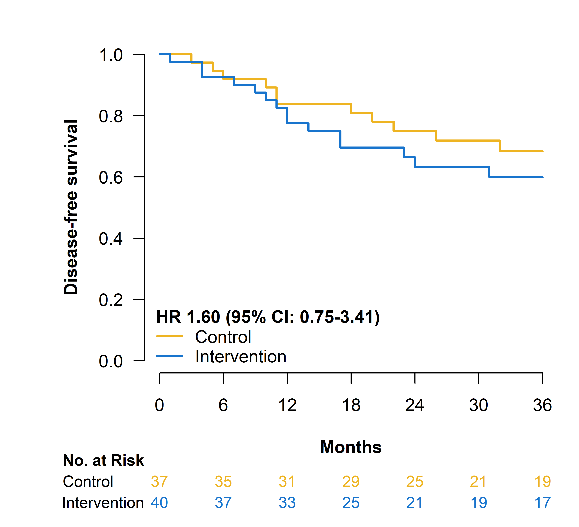


c. Overall survival


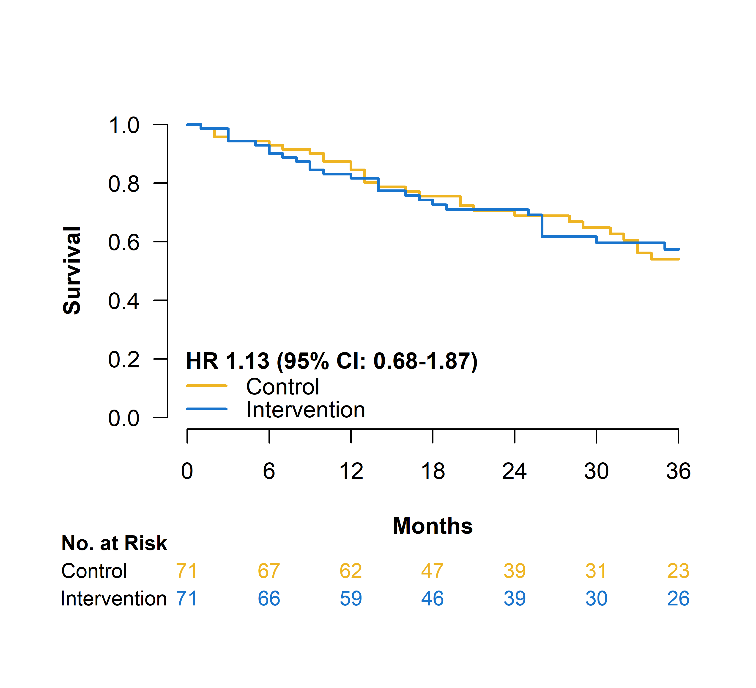


CI confidence interval; HR hazard ratio

b. Progression-free survival


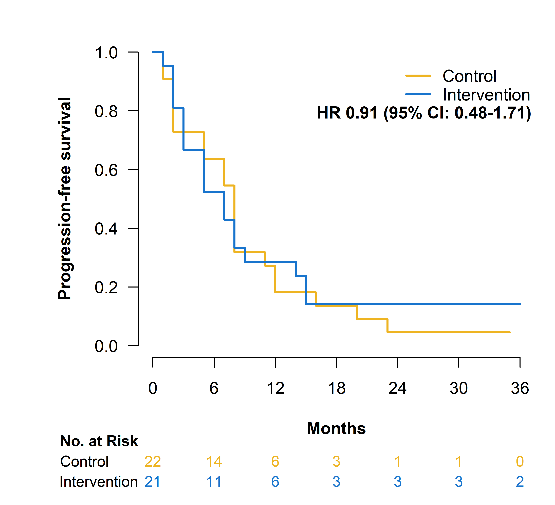


d. Colorectal cancer mortality


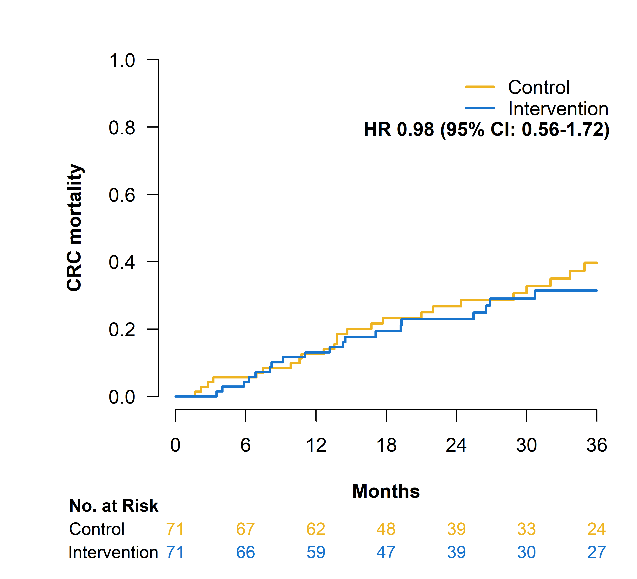


**Figure S2.**

a. Disease-free survival


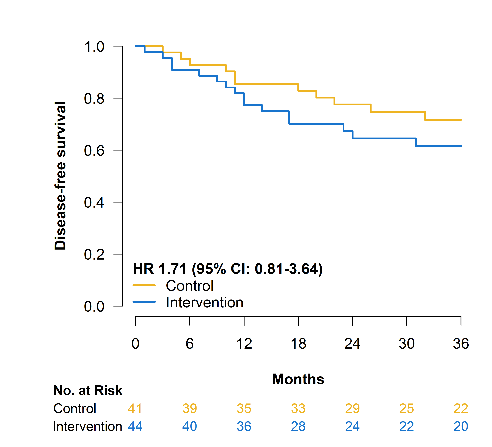


c. Overall survival


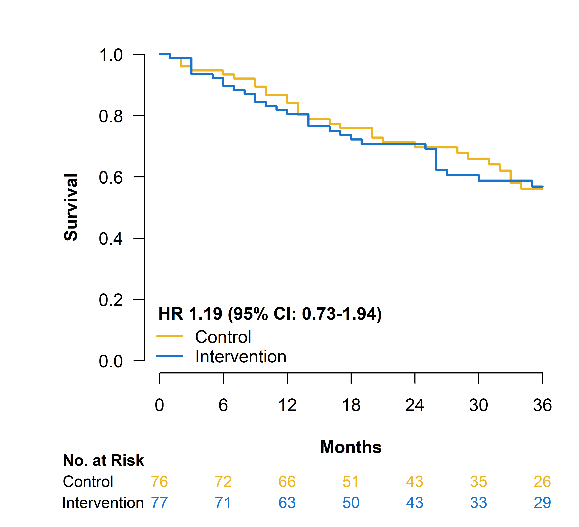


CI, Confidence interval; HR, Hazard ratio

b. Progression-free survival


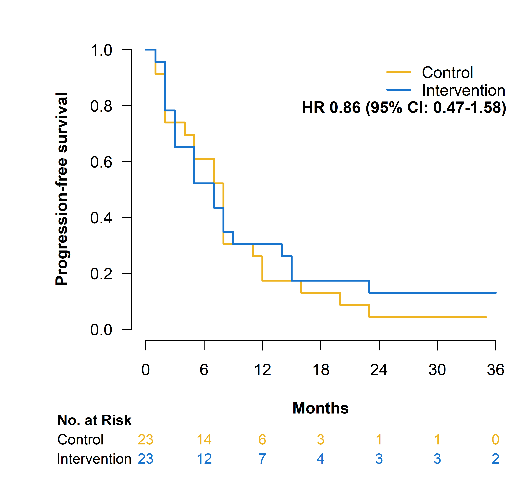


d. Colorectal cancer mortality


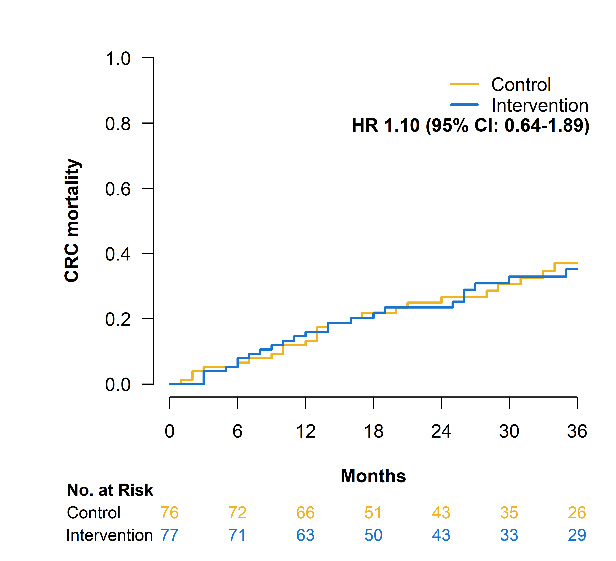


**Supplementary Figure Legends**

**Figure S1.** Disease-free survival for patients in adjuvant setting (a), Progression-free survival for patients in palliative setting (b), overall survival based on all patients (c), and CRC- mortality based on all patients (d), for patients in the intervention and control group.

**Figure S2**. Intention to treat analyses based on all 153 included patients.

Disease-free survival for patients in adjuvant setting (a), Progression-free survival for patients in palliative setting (b), overall survival based on all patients (c), and CRC- mortality based on all patients (d), for patients in the intervention and control group.
